# Supplementary material for: Quantifying the ecological carrying capacity of grasslands in Inner Mongolia
Source: PLoS One. 2023 Nov 22;18(11):e0291381. doi: 10.1371/journal.pone.0291381 (PMC10664909; doi:10.1371/journal.pone.0291381)
Supplement: S1 Raw images — (DOCX) [file pone.0291381.s001.docx]

**Figure legends**

**Fig 1 The study area and the sampling plots.**  The red triangles are the sampling plots in the field. Line is the boundary of the study area. The green areas are the grassland in Inner Mongolia. IA is the abbreviation of the Cold temperate humid region, IIA is the abbreviation of the Medium temperate humid region, IIB is the abbreviation of the Medium temperate sub-humid region, IIC is the abbreviation of the Medium temperate semi-arid region, IID is the abbreviation of the Medium temperate arid region, IIIB is the abbreviation of the Warm temperate semi-humid region Legend.


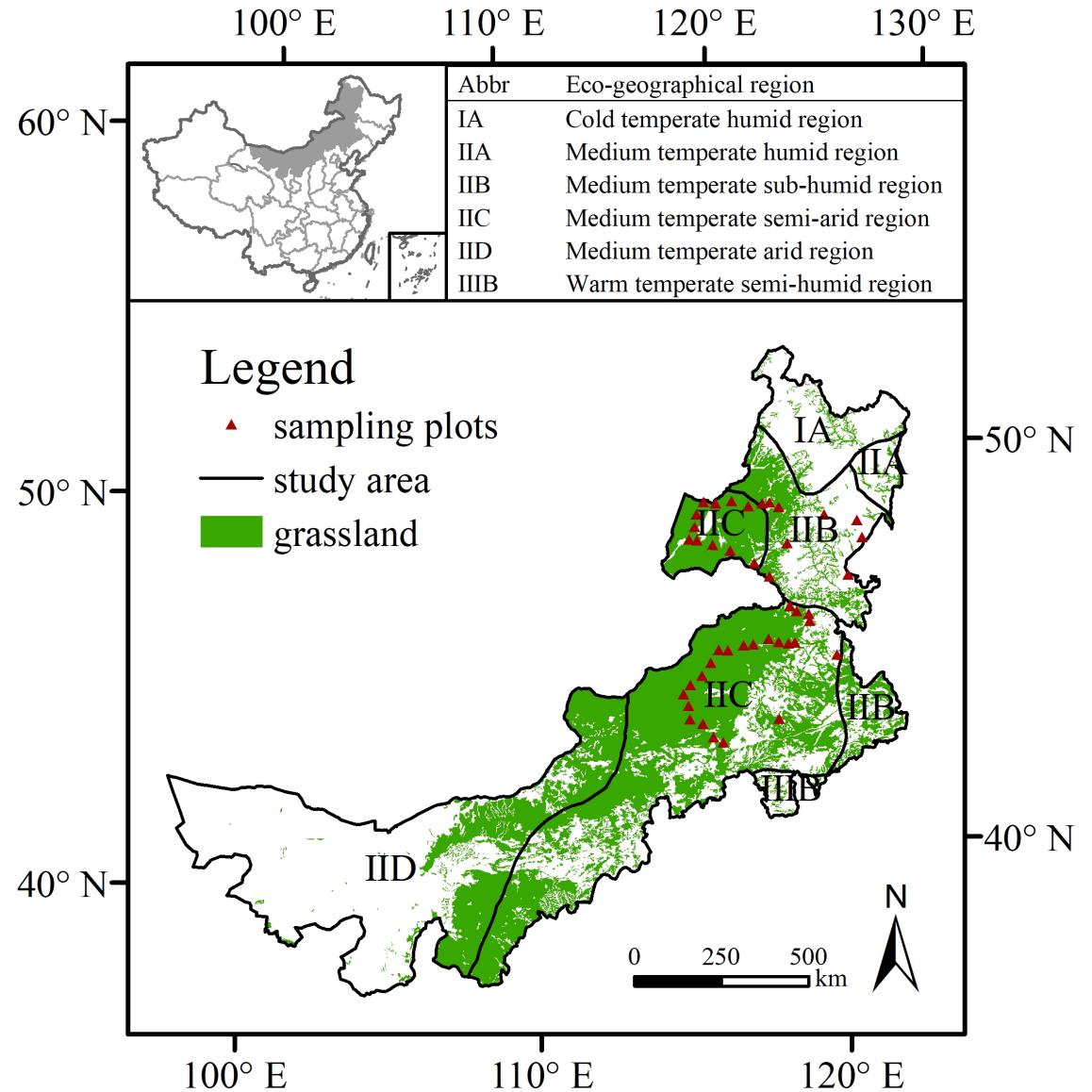


The image was obtained by using ArcGIS 10.2 through the open-access data process. The spatial extent of the Inner Mongolia was obtained from the Resource and Environmental Science Data Center of the Chinese Academy of Sciences (http://www.resdc.cn/).

**Fig 2.** **Spatial patterns of multi-year average (a) ANPP*_SC_*** (ANPP requires to support soil conservation from water erosion in the period 1987-2015), **(b) ANPP*_SF_*** (ANPP requires to support sand fixation from wind erosion in the period 1987-2015), **(c) ANPP*_NR_*** (ANPP requires to support natural regeneration in the period 1987-2015) and **(d) ANPP*_ES_*** (ANPP requires to support complete ecosystem services in Inner Mongolia grassland from 1987 to 2015).


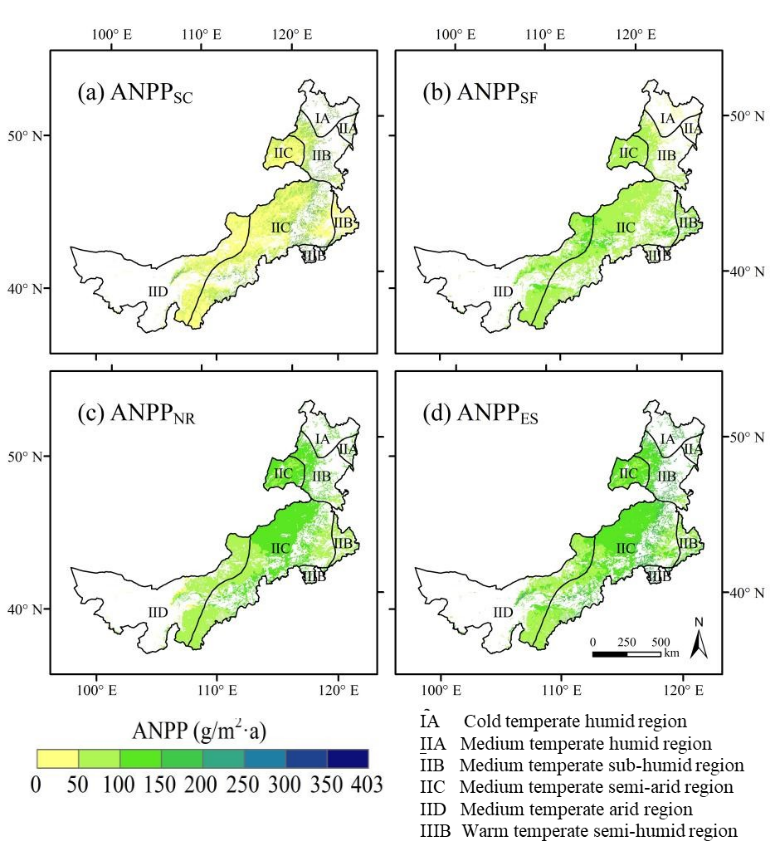


The image was obtained by using ArcGIS 10.2 through the open-access data process. The spatial extent of the Inner Mongolia was obtained from the Resource and Environmental Science Data Center of the Chinese Academy of Sciences (http://www.resdc.cn/). ANPP*_SC_* is derived from the Revised Universal Soil Loss Equation (RUSLE); ANPP*_SF_* is derived from the Revised Wind Erosion Equation (RWEQ); *ANPP_NR_* is the part of ANPP required to support the ES of natural regeneration, which is specified as 50% of ANPP; ANPP*_ES_* is the maximum ANPP of ANPP_SC,_ ANPP*_SF_*_,_ and *ANPP_NR._* Blank areas in (a), (b), (c) and (d) represent non-grassland areas.

**Fig 3.** **Spatial pattern of multi-year average ecological carrying capacity (ECC, sheep units hm^-2^year^-1^) in Inner Mongolia grassland from 1987 to 2015.** Blank areas represent non-grassland areas.


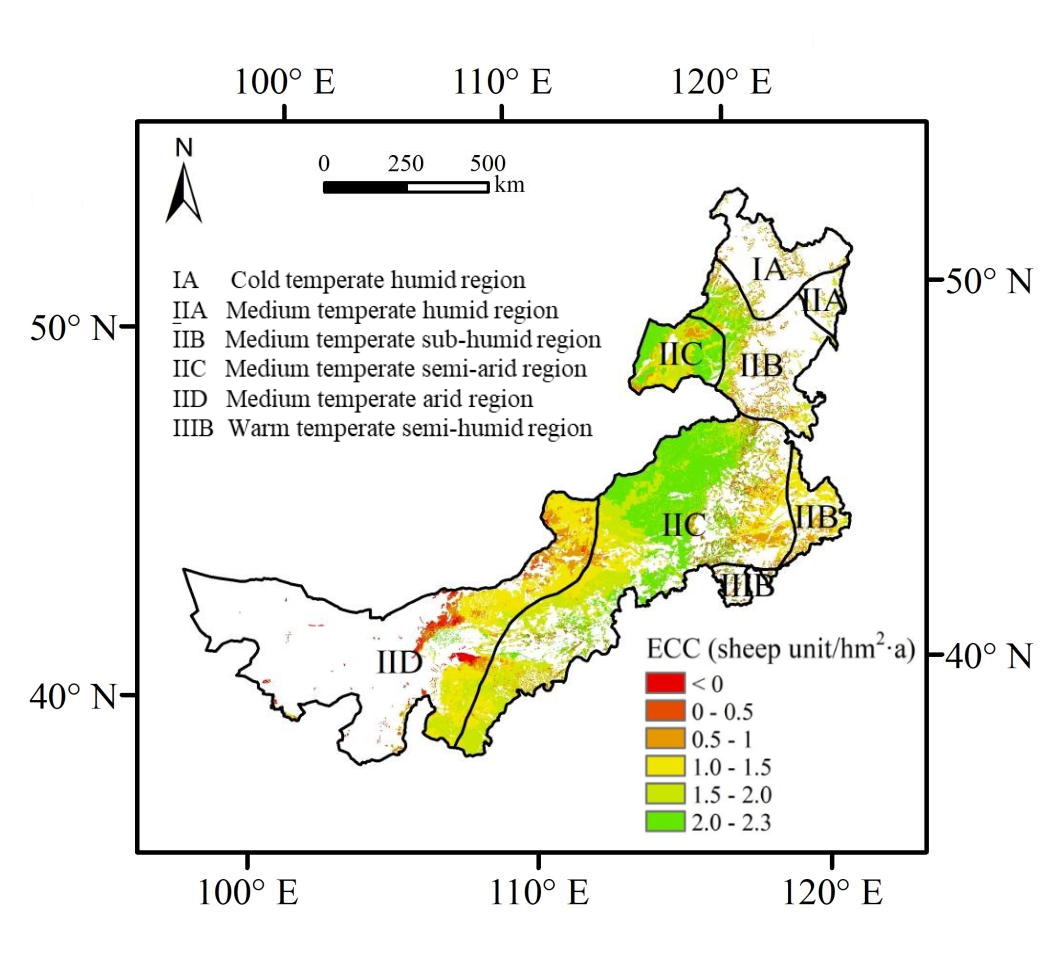


The image was obtained by using ArcGIS 10.2 through the open-access data process. The spatial extent of the Inner Mongolia was obtained from the Resource and Environmental Science Data Center of the Chinese Academy of Sciences (http://www.resdc.cn/).

**Fig 4.** ***q* values of influencing factors for different eco-geographical regions**
