# Supplementary material for: Quantifying the ecological carrying capacity of grasslands in Inner Mongolia
Source: PLoS One. 2023 Nov 22;18(11):e0291381. doi: 10.1371/journal.pone.0291381 (PMC10664909; doi:10.1371/journal.pone.0291381)
Supplement: S1 Table — (DOCX) [file pone.0291381.s002.docx]

# **Appendices**

**A list of acronyms**

| **Acronyms** | **Descriptions** | **Unites** |
| --- | --- | --- |
| *NPP* | Net primary production | gCm^-2^ |
| *ANPP* | Above-ground net primary production | gCm^-2^ |
| *ANPP_SC_* | ANPP required to support the ES of soil conservation | gCm^-2^ |
| *ANPP_SF_* | ANPP required to support the ES of sand fixation | gCm^-2^ |
| *ANPP_NR_* | ANPP required to support the ES of natural regeneration | gCm^-2^ |
| ${ANPP}_{ES}$ | The maximum ANPP of the all services | gCm^-2^ |
| *UNPP* | ANPP that can be used by animal husbandry | gCm^-2^ |
| ECC | Ecological carrying capacity | gCm^-2^ |

# **Supplementary methods description**

## Estimation of grassland ANPP

Grassland AGB was estimated based on the remote sensing inversion method of grassland biomass proposed by Gong (2017). Grassland height was estimated by using the classification and regression tree algorithm (CART). Then, the relationship between grassland AGB, grassland height and NDVI was established by regression analysis, expressed as follows:

$\begin{matrix} AGB=-292.292NDVI^{2}+357.377NDVI+3.811H-39.625 \\ (R^{2}=0.713,P<0.001) \end{matrix}$ (1)

AGB was converted to ANPP according to the ratio of ANPP to AGB (Ma *et al*., 2010) with the following formula:

$ANPP=1.092AGB$ (2)

## Estimation of ANPP_SC_, ANPP_SF_

The Revised Universal Soil Loss Equation (RUSLE) (Renard *et al*. 1997) was used to calculate the minimum vegetation coverage required to prevent soil erosion by water below a threshold (VC_SC_). The RUSLE model is described as follows:

 (3)

$C=\frac{A}{R\cdot K\cdot LS\cdot P}$ (4)

where *A* is the threshold of soil loss caused by water erosion (t hm^-2^·yr^-1^), While the threshold of soil water erosion was set as 5 t hm^-2^yr^-1^ according to “Water Conservancy Industry Standard of the People's Republic of China (SL190-2007)”; *R* is the rainfall erosivity factor (MJ·mm/hm^-1^·h^-1^·yr^-1^); *K* is the soil erodibility factor (t·h MJ^-1^ mm^-1^); *LS* is the factor of slope length and steepness; *C* is the vegetation coverage factor; *P* is erosion control practice factor.

the effective vegetation cover required to make soil water erosion lower than the threshold of soil loss (*EVC_water_*) was calculated as follows:

${EVC}_{water}=\left\{ \begin{aligned} {10}^{\frac{0.6508-C}{0.3436}}, 0\leq C<1 \\ 0, C=1 \end{aligned} \right.$ (5)

$R_{i}$ was calculated as (Sun *et al*., 2020):

$R_{i}=a\sum_{j=1}^{k} \left( D_{j} \right)^{\beta}$ (6)

$\beta=0.8363+\frac{18.144}{P_{d12}}+\frac{24.455}{P_{y12}}$ (7)

$\alpha=21.586 \beta^{-7.1891}$ (8)

Where $R_{i}$ is the R value of the i month (MJ mm hm -1h-1); k is the number of days in the i month; and $D_{j}$ is the effective precipitation for day j of the i month, which is no ＜12 mm. Otherwise, $D_{j}$ is equal to 0. $\alpha$ and $\beta$ are the parameters; d12 is the average daily rainfall (＞12 mm), and y12 is the yearly average rainfall for days with rainfall ＞12 mm. Annual R is aggregated by R value of each month.

And the parameters of K, LS, and P were calculated as (Yang *et al*. 2022).

The Revised Wind Erosion Equation (RWEQ) (Fryrear 2000) was adopted to calculate the minimum VC required to prevent soil erosion by wind below a threshold. The RWEQ model involved basic equations as follows:

$S_{L}=\frac{2z}{S^{2}}Q_{max}e^{-(z/S)^{2}}$ (9)

$S=105.71(WF\cdot EF\cdot SCF\cdot K'\cdot COG)^{-0.3711}$ (10)

$Q_{max}=109.8 (WF\cdot EF\cdot SCF\cdot K^{'}\cdot COG)$ (11)

where *S_L_* is the annual soil loss caused by wind erosion (kg m^-2^·yr^-1^),while soil loss tolerance was set as 2 t hm^-2^ yr^-1^ according to “Water Conservancy Industry Standard of the People's Republic of China (SL190-2007)”; *Q_max_* is the maximum transport capacity (kg m^-1^); *z* is the distance from the upwind edge of a field (m); *S* is the critical field length (m); *WF* is the weather factor (kg m^-1^); *EF* is the soil erodibility factor; *SCF* is the soil crust factor; *K'* is the soil roughness factor; *COG* is vegetation factor.
