# Supplementary material for: Quantifying the ecological carrying capacity of grasslands in Inner Mongolia
Source: PLoS One. 2023 Nov 22;18(11):e0291381. doi: 10.1371/journal.pone.0291381 (PMC10664909; doi:10.1371/journal.pone.0291381)
Supplement: Attachment — Submitted filename: Responses to reviewers.docx [file pone.0291381.s004.docx]

Dear editor Liu and reviewers:

We would like to express our great appreciation to you for your efforts in the previous version of the manuscript. You gave very valuable suggestions and comments for the paper which are of great help for us to improve our manuscript. We have studied reviewer’s comments carefully. According to the reviewer’s detailed suggestions, we have made a careful revision on the original manuscript. All revised portions are marked using track changes. Here we would like to submit the revised version comply with the PLOS ONE submission guidelines. We hope that the revised manuscript is satisfactory to your journal.

**Journal Requirements:**

When submitting your revision, we need you to address these additional requirements.

1. Please ensure that your manuscript meets PLOS ONE's style requirements, including those for file naming. The PLOS ONE style templates can be found at https://journals.plos.org/plosone/s/file?id=wjVg/PLOSOne_formatting_sample_main_body.pdf and https://journals.plos.org/plosone/s/file?id=ba62/PLOSOne_formatting_sample_title_authors_affiliations.pdf.

Response: Thank you for your comments. We have modified the manuscript as the PLOS ONE style templates (Details are in the “Revised Manuscript with Track Changes”).

 2. We suggest you thoroughly copyedit your manuscript for language usage, spelling, and grammar. If you do not know anyone who can help you do this, you may wish to consider employing a professional scientific editing service.

Upon resubmission, please provide the following: 
● The name of the colleague or the details of the professional service that edited your manuscript.
● A copy of your manuscript showing your changes by either highlighting them or using track changes (uploaded as a *supporting information* file).
● A clean copy of the edited manuscript (uploaded as the new *manuscript* file).

Response: Thank you very much for your professional recommendation. The manuscript has been edited by professional editors Vipin at Editage to ensure language and grammar accuracy. The logical presentation of ideas and the structure of the paper were also checked during the editing process. The author's core research ideas were not altered during the editing process.

3. In your Methods section, please provide additional information regarding the permits you obtained for the work. Please ensure you have included the full name of the authority that approved the field site access and, if no permits were required, a brief statement explaining why.

 Response: I apologize for the oversight in not sufficiently including the permission details for the AGB sampling. Your gentle reminder is valued. In the Methods section, we obtained the AGB data from a field survey carried out in Inner Mongolia at the peak of grassland biomass. Given that our surveys were conducted in spacious, unobtrusive grasslands with negligible environmental impact, no permits were necessary.

4. Thank you for stating the following financial disclosure: 
   "Scientific research Project of Education Department of Hunan Province (21C0744) "

Please state what role the funders took in the study.  If the funders had no role, please state: "The funders had no role in study design, data collection and analysis, decision to publish, or preparation of the manuscript." If this statement is not correct you must amend it as needed. Please include this amended Role of Funder statement in your cover letter; we will change the online submission form on your behalf.

Response: Thank you for your comments. We have stated the funders and its role in this study in our cover letter: “This study was supported by the Scientific Research Foundation of the Hunan Provincial Education Department (21C0744). The funder had no role in study design, data collection and analysis, the decision to publish, or the preparation of the manuscript.” (Line 36-38).

5. In your Data Availability statement, you have not specified where the minimal data set underlying the results described in your manuscript can be found. PLOS defines a study's minimal data set as the underlying data used to reach the conclusions drawn in the manuscript and any additional data required to replicate the reported study findings in their entirety. All PLOS journals require that the minimal data set be made fully available. For more information about our data policy, please see http://journals.plos.org/plosone/s/data-availability.

"Upon re-submitting your revised manuscript, please upload your study minimal underlying data set as either Supporting Information files or to a stable, public repository and include the relevant URLs, DOIs, or accession numbers within your revised cover letter. For a list of acceptable repositories, please see http://journals.plos.org/plosone/s/data-availability#loc-recommended-repositories. Any potentially identifying patient information must be fully anonymized.

Important: If there are ethical or legal restrictions to sharing your data publicly, please explain these restrictions in detail. Please see our guidelines for more information on what we consider unacceptable restrictions to publicly sharing data: http://journals.plos.org/plosone/s/data-availability#loc-unacceptable-data-access-restrictions. Note that it is not acceptable for the authors to be the sole named individuals responsible for ensuring data access.

We will update your Data Availability statement to reflect the information you provide in your cover letter.

Response: Thank you for your comments. We have uploaded the data as “supporting information”.

6. We note that Figures 1-3 in your submission contain [map/satellite] images which may be copyrighted. All PLOS content is published under the Creative Commons Attribution License (CC BY 4.0), which means that the manuscript, images, and Supporting Information files will be freely available online, and any third party is permitted to access, download, copy, distribute, and use these materials in any way, even commercially, with proper attribution. For these reasons, we cannot publish previously copyrighted maps or satellite images created using proprietary data, such as Google software (Google Maps, Street View, and Earth). For more information, see our copyright guidelines: http://journals.plos.org/plosone/s/licenses-and-copyright. 
We require you to either (a) present written permission from the copyright holder to publish these figures specifically under the CC BY 4.0 license, or (b) remove the figures from your submission:
 a. You may seek permission from the original copyright holder of Figures 1-3 to publish the content specifically under the CC BY 4.0 license.   
We recommend that you contact the original copyright holder with the Content Permission Form (http://journals.plos.org/plosone/s/file?id=7c09/content-permission-form.pdf) and the following text: “I request permission for the open-access journal PLOS ONE to publish XXX under the Creative Commons Attribution License (CCAL) CC BY 4.0 (http://creativecommons.org/licenses/by/4.0/). Please be aware that this license allows unrestricted use and distribution, even commercially, by third parties. Please reply and provide explicit written permission to publish XXX under a CC BY license and complete the attached form.” Please upload the completed Content Permission Form or other proof of granted permissions as an "Other" file with your submission. In the figure caption of the copyrighted figure, please include the following text: “Reprinted from [ref] under a CC BY license, with permission from [name of publisher], original copyright [original copyright year].” b. If you are unable to obtain permission from the original copyright holder to publish these figures under the CC BY 4.0 license or if the copyright holder’s requirements are incompatible with the CC BY 4.0 license, please either i) remove the figure or ii) supply a replacement figure that complies with the CC BY 4.0 license. Please check copyright information on all replacement figures and update the figure caption with source information. If applicable, please specify in the figure caption text when a figure is similar but not identical to the original image and is therefore for illustrative purposes only. The following resources for replacing copyrighted map figures may be helpful: 
USGS National Map Viewer (public domain): http://viewer.nationalmap.gov/viewer/The Gateway to Astronaut Photography of Earth (public domain): http://eol.jsc.nasa.gov/sseop/clickmap/Maps at the CIA (public domain): https://www.cia.gov/library/publications/the-world-factbook/index.html and <https://www.cia.gov/library/publications/cia-maps-publications/index.html> NASA Earth Observatory (public domain): http://earthobservatory.nasa.gov/Landsat: http://landsat.visibleearth.nasa.gov/USGS EROS (Earth Resources Observatory and Science (EROS) Center) (public domain): http://eros.usgs.gov/#Natural Earth (public domain): <http://www.naturalearthdata.com/>.

Response: Thank you for your comments. The images were obtained by using ArcGIS 10.2 through the open-access data process. The spatial extent of the Inner Mongolia was obtained from the Resource and Environmental Science Data Center of the Chinese Academy of Sciences (<http://www.resdc.cn/>). They are all the open-access data.

7. Please include a copy of Table 1 and 2 which you refer to in your text on pages 19 and 24.

Response: Thank you for your comments. We have added Tables 1 and 2 mentioned in the text to the manuscript (Line 262-263, 409-410).

8. Please include captions for your Supporting Information files at the end of your manuscript, and update any in-text citations to match accordingly. Please see our Supporting Information guidelines for more information: http://journals.plos.org/plosone/s/supporting-information.

Additional Editor Comments:
after reading the paper, the author should strengthen the statement of research novelty. meanwhile, the result and discussion analysis should be deepened. please revise the ms thoroughly.

Response: Thank you for your comments. We have included captions for our Supporting Information files at the end of the manuscript, and update the citations to match accordingly (Line 728-731).

We strengthen the statement of research novelty in the Introduction section (Line 134-157). And added the “Implications for grassland ecosystem sustainable management” section to analysis the research deeply (Line 452-476).

We have revised the manuscript thoroughly according the reviewer’s requirements.

**Comments to the Author**
Reviewer #1: This study presents a calculation framework for determining ecological carrying capacity in vulnerable areas using data from animal husbandry, soil conservation, sand fixation, and natural regeneration. The framework was applied to assess the ecological carrying capacity of Inner Mongolia (measured in sheep unit hm-2) and identify drivers of spatial heterogeneity. While the topic is interesting, the writing could benefit from further improvement. Therefore, I suggest a minor revision.
The introduction does not adequately support the novelty and importance of this study. Additional studies should be cited to demonstrate that previous research has overlooked correlations between environmental and social factors.
Response: Thank you for your comments. We cited the two papers to illustrate the importance of the harmonious coexistence of the natural environment and societal development.

[2] Ostad-Ali-Askari, K., Shayannejad, M., 2020. Impermanent changes investigation of shape factors of the volumetric balance model for water development in surface irrigation. Modeling Earth Systems and Environment. 6, 1573–1580.

[3] Nafchi, R.F., Samadi-Boroujeni, H., Vanani, H.R., Ostad-Ali-Askari, K., Brojeni, M.K., 2021. Laboratory investigation on erosion threshold shear stress of cohesive sediment in Karkheh Dam. Environ. Earth Sci. 80, 19.(Line 68-69).

We cited the paper of Ostad-Ali-Askari and Shayannejad (2021) to illustrate the importance of calculating the ECC :

[9] Ostad-Ali-Askari, K., Shayannejad, M., 2021a. Computation of subsurface drain spacing in the unsteady conditions using Artificial Neural Networks (ANN). Appl. Water Sci. 11 (2), 21 (Line 77).

We cited the three papers to demonstrate that previous research has overlooked correlations between environmental and social factors.

[15] Wu XL, Hu F. Analysis of ecological carrying capacity using a fuzzy comprehensive evaluation method. Ecological Indicators. 2020; 113: 106243. <https://doi.org/10.1016/j.ecolind.2020.106243> .

[16] Bu J, Li, C, Wang, X, Wang X, Zhang Y, Yang ZW. Assessment and prediction of the water ecological carrying capacity in Changzhou city, China. Journal of Cleaner Production. 2020; 277: 123-988. [https://doi.org/10.1016/j.jclepro.2020.123988](https://doi.org/10.1016/j.jclepro.2020.123988 ) .

[17] Thiesen T, Bhat MG, Liu H, Rovira R. An ecosystem service approach to assessing agro-ecosystems in urban landscapes. Land. 2022; 11 (4), 469. <https://doi.org/10.3390/land11040469> (Line 88-93).

The authors aim to propose an improved calculation framework for ecological carrying capacity, while they do not provide sufficient evidence supporting its advantages over existing methods. Comparing their results with those of other studies on spatial distribution would strengthen their argument.

Response: Thank you for your comments. There are relatively few literatures on the calculation of ecological carrying capacity of Inner Mongolia grassland. We have compared our result with other studies on spatial distribution in the “Comparison between ECC” section (Line 418-436).

Many abbreviations are unnecessary, such as ES, MA, IA IID and IIIB, which actually increase the difficulty of reading.
Response: Thank you for your comments. We have removed unnecessary abbreviations, such as ES, MA, IA, IIA, IIB, IIC, IID and IIIB.

Line 175-176: The formula layout is incorrect and needs further adjustment.
Response: Thank you for your comments. We have adjusted the formula layout (Line 208, 222-223).

Figure 4: The image needs to be self-explanatory. If abbreviations are used, they need to be explained in the Figure caption.

Response: Thank you for your comments. We have explained the abbreviations in the each Figure caption.

6. PLOS authors have the option to publish the peer review history of their article ([what does this mean?](https://journals.plos.org/plosone/s/editorial-and-peer-review-process#loc-peer-review-history)). If published, this will include your full peer review and any attached files.
If you choose “no”, your identity will remain anonymous but your review may still be made public.
Do you want your identity to be public for this peer review? For information about this choice, including consent withdrawal, please see our [Privacy Policy](https://www.plos.org/privacy-policy).

Reviewer #1: No

[NOTE: If reviewer comments were submitted as an attachment file, they will be attached to this email and accessible via the submission site. Please log into your account, locate the manuscript record, and check for the action link "View Attachments". If this link does not appear, there are no attachment files.]

Response: Thank you for your comments. Our choice is No.

While revising your submission, please upload your figure files to the Preflight Analysis and Conversion Engine (PACE) digital diagnostic tool, <https://pacev2.apexcovantage.com/>. PACE helps ensure that figures meet PLOS requirements. To use PACE, you must first register as a user. Registration is free. Then, login and navigate to the UPLOAD tab, where you will find detailed instructions on how to use the tool. If you encounter any issues or have any questions when using PACE, please email PLOS at [figures@plos.org](mailto:figures@plos.org). Please note that Supporting Information files do not need this step.

Response: Thank you for your comments. We have checked the figures to ensure they meet PLOS requirements.

Once again, thank you very much for your constructive comments and suggestions which would help us both in English and in depth to improve the quality of the paper.

Kind regards,

authors

E-mail: [guocy.17b@igsnrr.ac.cn](mailto:guocy.17b@igsnrr.ac.cn)
